# Does attendance at the ECTRIMS congress impact on therapeutic decisions in multiple sclerosis care?

**DOI:** 10.1177/2055217319835226

**Published:** 2019-03-18

**Authors:** Gustavo Saposnik, Jorge Maurino, Angel P Sempere, Maria A Terzaghi, Maria Pia Amato, Xavier Montalban

**Affiliations:** Department of Medicine, St. Michael’s Hospital, Toronto, Canada; Li Ka Shing Knowledge Institute, St. Michael’s Hospital, Toronto, Canada; Department of Economics, University of Zurich, Switzerland; University of Buenos Aires, Argentina; Medical Department, Roche Farma, Madrid, Spain; Department of Neurology, Hospital General Universitario de Alicante, Spain; Li Ka Shing Knowledge Institute, St. Michael’s Hospital, Toronto, Canada; NEUROFARBA Department, Neurosciences Section, University of Florence, Italy; IRCCS Fondazione Don Carlo Gnocchi, Florence, Italy; Department of Medicine, St. Michael’s Hospital, Toronto, Canada; Department of Neurology-Neuroimmunology, Hospital Universitari Vall d´Hebron, Barcelona, Spain

**Keywords:** Continuing medical education, management errors, behavioral economics, medical decisions, multiple sclerosis, ECTRIMS

## Abstract

Conferences traditionally play an important role in the ongoing medical education of healthcare professionals. We assessed the influence of attending the ECTRIMS congress on therapeutic decision-making in multiple sclerosis (MS) care. A non-interventional, cross-sectional study involving 96 neurologists was conducted. Treatment escalation when therapeutic goals were unmet and management errors related to tolerability and safety scenarios of MS therapies were tested using different case-scenarios. Attendance at ECTRIMS was associated with an increase likelihood of treatment escalation in the presence of clinical progression (cognitive decline) and radiological activity (OR 2.44; 95% CI 1.06–5.82) and lower number of management errors (OR 0.26; 95% CI 0.07–0.98). Attendance at ECTRIMS may facilitate therapeutic decisions and reduction in management errors in MS care.

## Introduction

Continuing medical education (CME) is a key part of postgraduate training for healthcare professionals (HCP) to gain knowledge that ensures optimal care and outcomes for patients.^1,2^ Medical conferences traditionally play an important role in the ongoing medical education of HCP, providing access to breaking evidence from around the world.^3,4^

Making therapeutic decisions in multiple sclerosis (MS) is becoming increasingly difficult due to the more complicated risk–benefit spectrum of new disease-modifying therapies (DMTs).^5,6^ The European Committee for Treatment and Research in Multiple Sclerosis (ECTRIMS) is a non-profit organization created in 1985 to promote research and learning among professionals involved in the management of people with MS.^7^ At the annual ECTRIMS congress, up to 10,000 participants have the opportunity to discuss the latest scientific research. However, limited information is available on the impact of attending the ECTRIMS congress on the management of patients with MS. The aim of this study was to assess the influence of attending the last ECTRIMS congress on therapeutic decisions and management errors by applying principles from behavioral economics.

## Methods

A non-interventional, cross-sectional, web-based study using the Qualtrics platform (http://qualtrics.com) was conducted (*DIScUTIR MS Study*).^8,9^ The aim of this study was to evaluate whether neurologists’ risk preferences were associated with therapeutic inertia in MS care. We implemented a novel approach combining case-vignettes with the assessment of cognitive biases through validated experiments in behavioral economics.^6,9^ The application of these principles may help overcome those barriers by identifying and increasing awareness about cognitive biases or risk preferences (e.g. overconfidence, tolerance to risk, ambiguity, etc.) that may lead to suboptimal decisions. A post-hoc analysis using data from the aforementioned study was performed by comparing therapeutic decisions between participants who attended versus those who did not attend ECTRIMS (exposure). Practicing neurologists actively involved in the care of patients with MS from across Spain were invited to participate in the study by the Spanish Society of Neurology (Sociedad Española de Neurología-SEN). Participants were exposed to 20 simulated MS case-scenarios, three standardized surveys, and four behavioral experiments to assess aversion to risk and ambiguity (unknown probability of an event). Of the 20 simulated case-scenarios, seven scenarios were designed to determine the presence of therapeutic inertia with evidence of recurrent clinical relapses and radiological progression despite first line therapies. Three case scenarios were designed to assess the appropriate management of side effects of therapies (e.g. transaminitis, lymphopenia, and gastrointestinal side effects). The remaining cases were designed to learn about physicians’ therapeutic preferences and are not accounted for in this analysis. Further details of the protocol were published elsewhere.^8^ Informed consent was obtained from all participants and the study was approved by the institutional review board of the St. Michael´s Hospital (Toronto, Canada).

## Study outcomes and definitions

We assessed treatment escalation when therapeutic goals were unmet (e.g. clinical and radiological evidence of disease progression) as defined in our previous studies.^8,9^ We completed two different analyses: (i) all case-scenarios and (ii) case-scenarios having a before and after cognitive testing (e.g. a Symbol Digit Modalities Test drop from over 60 to 40) showing a progressive cognitive decline plus evidence of disease progression by magnetic resonance imaging (e.g. at least five new/enlarging T2 lesions plus one or more gadolinium-enhancing T1 lesions).^8,10^

The outcome of interest was therapeutic inertia (TI) defined as a dichotomous variable (present if identified in at least two case-scenarios) and as a continuous variable (by the TI score defined according to the number of case-scenarios where participants exhibited inertia).^9^ A higher TI score indicates higher TI.

Management errors were tested with tolerability and safety scenarios of DMTs (e.g. transaminitis, lymphopenia, and gastrointestinal side effects).^11^ Mixed effects models were used to determine the association between TI score and TI with independent variables. All multivariable analyses were adjusted for age, level of expertise (specialty, practice setting, years of practice), and MS patient volume/week, and reported as odds ratio (OR) and 95% confidence interval (CI).

## Results

A total of 96 neurologists were included in the study. The main characteristics of the study population are shown in Table 1. The mean (±SD) age was 40 (±8.5) years and 51 (53.1%) were female neurologists.

**Table 1.** Baseline characteristics of participants.

Characteristics	Total*n* = 96	Attendees at ECTRIMS*n* = 56		Non-attendees*n* = 40	*p*-value
Age (mean ± SD), in years	39.5 ± 8.5	39.8 ± 8.5		39.3 ± 8.6	0.78
Age >40, in years	56 (58.3)	24 (42.9)		32 (57.1)	0.83
Gender, *n* (%)					
Female	51 (53.1)	32 (57.1)		19 (47.5)	0.35
MS expertise, *n* (%)					0.003
General neurologist	32 (33.3)	12 (21.4)		20 (50.0)	
MS specialist	64 (66.7)	44 (78.6)		20 (50.0)	
Practice setting, *n* (%)					0.56
Academic	69 (71.9)	39 (69.6)		30 (75.0)	
Community	27 (27.1)	17 (30.4)		10 (25)	
Years in practice, mean ± SD	14.1 ± 10	14.8 ± 11		13.1 ± 8	0.41
MS patients seen per week, mean ± SD	20 ± 15	22.8 ± 21		15.2 ± 13	0.05
Author of a peer-reviewed publication in the last 3 years, *n* (%)	79 (82.3)	49 (87.5)		30 (75.0)	0.11
Participants’ risk preferences					
Risk aversion^a^	26 (27.1)	17 (30.4)		9 (22.5)	0.39
Aversion to ambiguity^b^	26 (27.1)	15 (26.8)		11 (27.5)	0.94

Numbers between brackets represent percentages, unless otherwise specified.

^a^Participants choose a safe amount of 120 euros or less instead of a 50/50 chance of winning 400 euros.

^b^Participants choose the 50/50 known probability of winning 400 euros over the unknown probability of winning 400 euros. Further details are explained elsewhere.^8^

### Therapeutic inertia (TI)

Lack of treatment escalation was detected in at least one case-scenario in 68.8% of participants. The mean (±SD) TI score was 1.5 (±1.0).

The multilevel mixed-effects linear regression analysis revealed that participants who attended ECTRIMS had significantly lower TI scores (β coefficient −0.30, 95% CI −0.59 to −0.015; *p* = 0.039). The multilevel mixed-effects logistic regression analysis (TI as a dichotomous outcome) revealed that participants who attended ECTRIMS had 70% reduction (not reaching significance) in TI (OR 0.32; 95% CI 0.08–1.31).

Finally, the multivariable mixed effects model for case-scenarios with progressive cognitive decline plus radiological activity revealed that attendance at ECTRIMS was associated with an increased likelihood of treatment escalation (OR 2.44; 95% CI 1.06–5.82). There were no differences between fixed- and random-effects models.

### Medical management of side effects of DMTs

**Figure 1.**
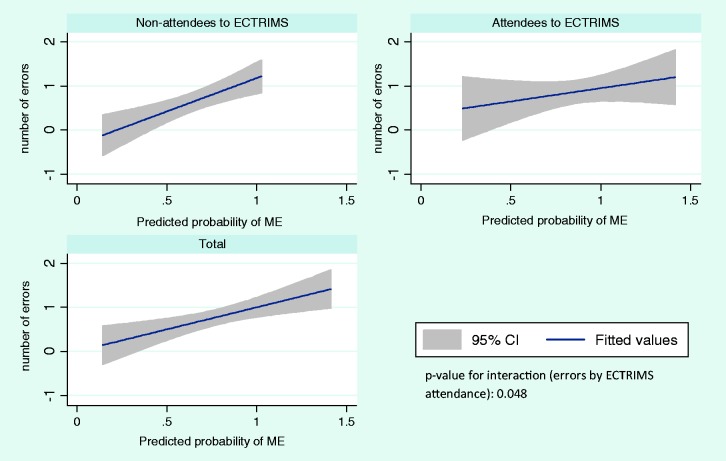
Predicted number of management errors (ME) by ECTRIMS attendance. Note differences in the slope of ME between attendees vs. non-attendees (*p* = 0.048).

One third of neurologists made at least one management error, whereas 18.8% made two errors out of three case-scenarios. The multivariable mixed effects model revealed the attendance to ECTRIMS was associated a lower number of management errors (OR 0.26; 95% CI 0.07–0.98). Figure 1 represents the predicted probability of management errors by ECTRIMS attendance after adjustment for covariates (*p*-value for interaction ECTRIMS attendance by management errors: 0.048). There was no association between participants risk preferences (e.g. risk aversion and aversion to ambiguity) with the outcomes of interest.

## Discussion

CME is especially relevant due to rapidly evolving knowledge and is a required element of maintenance of certification in most countries.^2,4^ CME has a positive impact on physician´s knowledge and performance.^3^ We found that participants who attended ECTRIMS were 2.5 times more likely to escalate treatment when there was evidence of disease activity and had a significant lower TI and lower number of management errors.

Previous studies found that didactic sessions did not appear to be effective in changing physician performance in a review of 14 randomized controlled studies of formal educational interventions including conferences, meetings, and symposia.^12^ Later on, Forsetlund et al. examined the effects of continuing education meetings on professional practice and patient outcomes.^13^ They reviewed 81 trials involving more than 11,000 HCP and found that higher attendance at educational meetings was associated with larger improvements in clinical practice. However, educational meetings did not appear to be effective for complex behaviors compared to less complex behaviors as well as less effective for less severe outcomes than for more serious ones.^13^

CME has evolved from a passive, traditional didactic approach to an interactive earner-centered approach involving new technologies. HCP can now get faster access to the information they need.^2^ Unfortunately, little data are available about effective educational interventions that target neurologists.^1^

Our study has several limitations that deserve comment. First, we included neurologists only from Spain, limiting the generalizability of our results. Second, we cannot rule out the role of unmeasured confounders (e.g. infrastructure of centers, differences in previous medical education, previous participation in different MS/general neurology conferences and/or CME resources other than ECTRIMS) and possible selection bias to explain our findings. Third, it is possible the presence of residual confounding despite the adjustment for relevant factors and differences in baseline characteristics. Finally, durability of the educational effect of attending this medical conference should be analyzed in future studies.

Our study suggests that attendance at ECTRIMS (the most well attended CME in the specialty) is associated with improved therapeutic decisions and reduction in management errors, confirming the positive role of CME to foster physicians’ knowledge and performance.

## Conclusion

ECTRIMS and possibly the attendance at other medical conferences may play a role as a complementary strategy to optimize long-term learning of neurologists that may facilitate therapeutic decisions and reduction in management errors in MS care.

## Note

The abstract of this paper was presented at the 34^th^ Congress of the European Committee for Treatment and Research in Multiple Sclerosis (ECTRIMS) as an eposter presentation with interim findings.
